# The Neural Basis of Risky Choice with Affective Outcomes

**DOI:** 10.1371/journal.pone.0122475

**Published:** 2015-04-01

**Authors:** Renata S. Suter, Thorsten Pachur, Ralph Hertwig, Tor Endestad, Guido Biele

**Affiliations:** 1 Center for Adaptive Rationality, Max Planck Institute for Human Development, Berlin, Germany; 2 Department of Psychology, University of Oslo, Oslo, Norway; 3 Norwegian Institute of Public Health, Oslo, Norway; Centre national de la recherche scientifique, FRANCE

## Abstract

Both normative and many descriptive theories of decision making under risk are based on the notion that outcomes are weighted by their probability, with subsequent maximization of the (subjective) expected outcome. Numerous investigations from psychology, economics, and neuroscience have produced evidence consistent with this notion. However, this research has typically investigated choices involving relatively affect-poor, monetary outcomes. We compared choice in relatively affect-poor, monetary lottery problems with choice in relatively affect-rich medical decision problems. Computational modeling of behavioral data and model-based neuroimaging analyses provide converging evidence for substantial differences in the respective decision mechanisms. Relative to affect-poor choices, affect-rich choices yielded a more strongly curved probability weighting function of cumulative prospect theory, thus signaling that the psychological impact of probabilities is strongly diminished for affect-rich outcomes. Examining task-dependent brain activation, we identified a region-by-condition interaction indicating qualitative differences of activation between affect-rich and affect-poor choices. Moreover, brain activation in regions that were more active during affect-poor choices (e.g., the supramarginal gyrus) correlated with individual trial-by-trial decision weights, indicating that these regions reflect processing of probabilities. Formal reverse inference Neurosynth meta-analyses suggested that whereas affect-poor choices seem to be based on brain mechanisms for calculative processes, affect-rich choices are driven by the representation of outcomes’ emotional value and autobiographical memories associated with them. These results provide evidence that the traditional notion of expectation maximization may not apply in the context of outcomes laden with affective responses, and that understanding the brain mechanisms of decision making requires the domain of the decision to be taken into account.

## Introduction

Traditional economic theory assumes that choices under risk rest on a trade-off between the options’ possible outcomes and their probabilities—that is, that outcomes (or some function of them) are weighted by how probable they are. This notion of *expectation maximization* is pervasive in the social and behavioral sciences, and underlies not only theories of risky decision making [1, 2], but also theories of moral judgment, work behavior, social learning, attitude formation, and health behavior [3–7].

Numerous psychological, economic, and neurobiological studies into risky decision making have provided evidence consistent with the notion of expectation maximization (though there is also contrary evidence) [8]. Most of these studies have asked people to evaluate options with relatively affect-poor outcomes—primarily, monetary lotteries [9–14]. Yet, many of the important decisions that people face—such as which potentially risky medical treatment to choose (e.g., chemotherapy) or whom to marry—have relatively affect-rich outcomes. Do these choices also rest on expectation maximization, and specifically, on the process of weighing of possible outcomes by their probabilities?

Several behavioral studies have identified differences in decision making between affect-rich (e.g., involving electric shocks) and affect-poor tasks [15, 16]. Using cumulative prospect theory [14], Rottenstreich and Hsee [17] argued that these differences could be accommodated by assuming a more strongly curved probability weighting function for affect-rich choices. A stronger curvature of the weighting function suggests that the psychological impact of probabilities is diminished, because a change on the probability dimension is not associated with a proportional change in the decision weight. An alternative account suggests that people in situations involving affect-rich risks tend to simply focus on avoiding the worst possible outcome and that, in the process of reaching this goal, the probability of occurrence takes a back seat [18–20]. A related switch in decision strategy—in which people reorient from focusing on probabilities in monetary gambles to focusing on outcomes (and, in particular, on avoiding meaningful potential losses)—has also been proposed for decision makers in whom positive affect has been induced [21]. In sum, the traditional notion of a mechanism that assumes sensitivity to outcome and probability information and expectation maximization may not hold when options elicit relatively high levels of affect. Instead, qualitatively different strategies may be used in affect-rich versus affect-poor decisions [20, 22].

Although these accounts make qualitatively different predictions about the neuro-cognitive mechanisms underlying affect-rich and affect-poor risky choice, they have yet to be tested against each other. Comparing choices in affect-rich and affect-poor problems, as well as the neural underpinnings of those choices, would test the generalizability of expectation maximization as well as the often implicit assumption that the neuro-cognitive mechanisms of risky choice are domain general. Moreover, tools for improving decision making in affect-rich contexts (e.g., a choice of medical treatments) may need to be designed differently depending on whether people are sensitive to the probabilities of rare but consequential outcomes (and potentially overweight them) or whether they do without the probability information and the respective weighting process. In what follows, we pit these two accounts against each other.

## Materials and Methods

In order to examine the neural underpinnings of cognitive processing in affect-rich and affect-poor decisions, we asked participants to make choices between two options with relatively affect-rich outcomes (drugs that cause a side effect with some probability) as well as between two options with relatively affect-poor outcomes (lotteries that incur monetary losses with some probability) [23, 24]. The monetary losses were matched to each individual’s subjective monetary evaluation of the side effects, permitting a within-subject comparison between affect-rich and affect-poor choices in otherwise monetarily equivalent problems.

### Subjects

Participants were 23 healthy subjects (mean age = 22.7 years, *SD* = 2.5, 14 female). The study was approved by the ethics committee of the University of Oslo’s Department of Psychology and was conducted at the University of Oslo (Oslo University Hospital). Participants reported no psychiatric or neurological disorders and gave their informed consent through a form approved by the ethics committee. They received a CD with images of their brain in appreciation of their participation. One participant had to be excluded from the fMRI data analysis due to scanning problems.

### Paradigm

**Fig 1:**
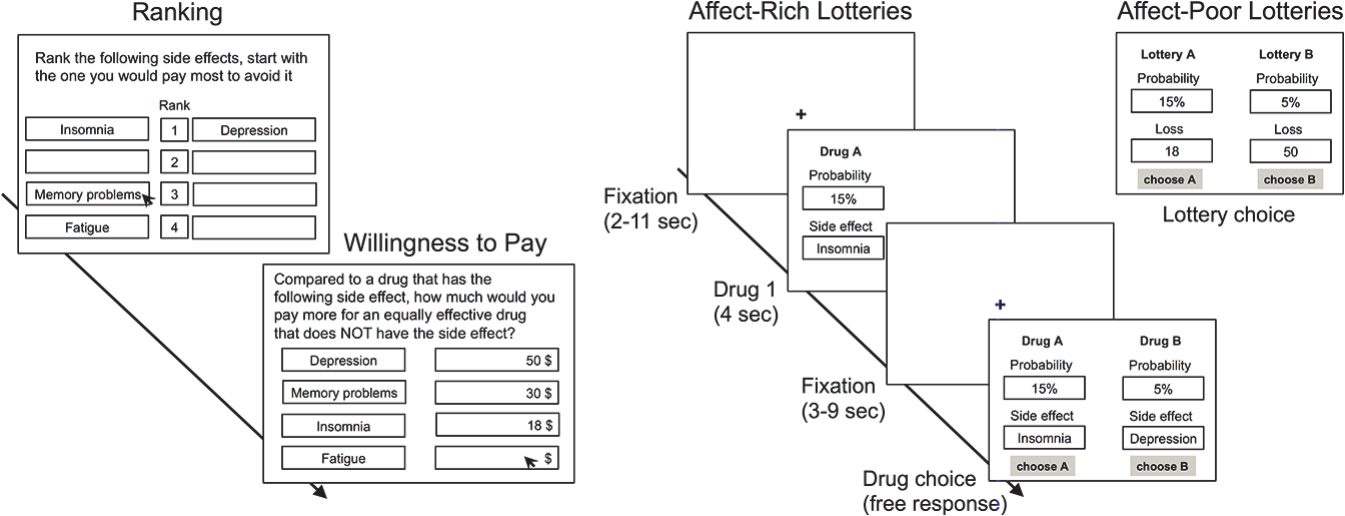
Monetary Evaluation Task and Lottery Problems. Experimental design. (A) Participants ranked four side effects of a drug from most to least unpleasant; they then indicated their willingness-to-pay (WTP) to avoid each of these side effects. (B) Next, participants were presented with affect-rich and affect-poor lottery problems, in which they were asked to choose between two options. In the affect-rich problems (84 choices), they were presented with two equally effective drugs, each with some probability of a side effect (e.g., insomnia with 15%). At each trial, one of the drugs was presented first (for 4 seconds; evaluation phase). After an interval of 2–9 seconds, both drugs were presented together, and participants indicated their choice (decision phase). In the affect-poor problems (84 choices), participants were shown the same decision problems as in the affect-rich problems, but with the side effects being replaced by each participant’s WTP.

As in other studies on affective decision making under risk [15, 17, 19, 25], we used nonmonetary outcomes to represent affect-rich stimuli—specifically, adverse medical side effects. Monetary losses (which were matched to the side effects in terms of what individual participants viewed as their economic equivalents, see below) were used as affect-poor outcomes. In studies using the same material [23, 24], participants indicated that they would be more upset if they experienced a side effect than if they had to pay the monetary amount they indicated as being economically equivalent to that side effect. Following previous research, we thus refer to outcomes as “affect-rich” and “affect-poor” in relative (rather than absolute) terms. Our paradigm was modeled on an approach developed by Pachur et al. [23, 24]: Participants were first asked to indicate the amount of money they considered equivalent to specific nonmonetary outcomes (here: side effects; Fig 1A). The monetary amounts indicated (willingness-to-pay; WTP) were then used to construct individualized lotteries in which either a side effect (affect-rich problem) or a monetary loss (affect-poor problem) occurred with some probability. For example, consider a participant who specified a WTP of $18 to avoid insomnia and $50 to avoid depression. In the affect-rich problem, she would be presented with a choice between drug A, leading to insomnia with a probability of 15% (no side effects otherwise), and drug B, leading to depression with a probability of 5% (no side effects otherwise). In the corresponding affect-poor problem, she would be presented with a choice between lottery A, leading to a loss of $18 with a probability of 15% (nothing otherwise), and lottery B, leading to a loss of $50 with a probability of 5% (nothing otherwise). This paradigm allowed us to compare the decision mechanisms underlying affect-rich versus affect-poor risky choice on the basis of lottery problems that were equivalent in monetary terms (Fig 1A and 1B).

### Materials

We used the following four side effects as affect-rich outcomes: fatigue, insomnia, depression, and memory problems. Participants were presented with three tasks: a monetary evaluation task, a choice task (involving affect-poor and affect-rich lottery problems), and an affective evaluation task. In the *monetary evaluation task*, participants first ranked the four side effects from most to least unpleasant (Fig 1A). Next, they indicated their willingness to pay (WTP; in Norwegian kroner) in order to avoid each of the side effects. Specifically, they were asked to imagine that they were suffering from an (unspecified) illness, needed to take a drug for one week, and that two possible drugs were available. Both treated the illness equally well, but one was certain to cause a particular side effect during the week of treatment (e.g., insomnia), whereas the other caused no side effects. Participants then indicated the amount of money they would be willing to pay as a markup for the drug without the side effect.

Next, participants were presented with a *choice task*, consisting of both affect-rich and affect-poor decision problems. In the former, they chose between two drugs, each potentially causing a particular side effect with some probability (see example above and Fig 1B). In the latter, they chose between two monetary lotteries, each presenting the prospect of losing a particular amount of money with some probability. As described above, we constructed these affect-poor problems on the basis of the affect-rich problems, replacing the side effects with the individual-specific WTPs obtained in the monetary evaluation task. As a result, the affect-poor problems were equivalent to the affect-rich ones in terms of monetary amounts and probabilities. Each problem involved a choice between two options (drugs or monetary lotteries), with one option implicating a less severe, but more probable, outcome than the other.

We used four probability levels (low, low-to-medium, medium, and high), with two probabilities representing each level (differing by 0.03), yielding a total of 8 probabilities: 0.05, 0.08, 0.15, 0.18, 0.5, 0.53, 0.95, and 0.98. Using these probabilities and the median WTPs obtained in a pilot study, we constructed 42 lottery problems consisting of pairs of lotteries with comparable expected values (see S1 Table for a complete list of the problems used. Note that each participant was presented with lottery problems containing her own WTPs; the median WTPs from the pilot study were used only to construct lottery problems with similar expected values). The decision problems in each domain were presented twice (see below), yielding a total of 84 affect-rich and 84 affect-poor problems.

Finally, participants were presented with a two-part *affective evaluation task*. First, each person was asked to imagine that she had lost a bet and would therefore suffer the loss of a specified amount of money. For each of the monetary amounts that a participant had indicated as WTPs in the monetary evaluation task, she now indicated, on a scale from 1 (= not upset) to 10 (= very upset), how upset she would be to suffer this loss. Second, she was asked to imagine that she needed to take a drug and would experience a side effect. For each side effect, she indicated how upset she would be to experience that effect.

### Design and Procedure

Participants were randomly assigned to one of two between-subjects conditions to control for order effects (see below). They first completed the monetary evaluation task outside the scanner. In the scanner, they were then asked to render choices between both affect-rich and affect-poor options. The choice task was spread across three scanning sessions. No feedback concerning trial outcomes was given. Each trial in the choice task started with an *evaluation phase*, where one option was displayed for 4 seconds (panel B in Fig 1) [26]. In the subsequent *decision phase*, both options were displayed, and participants were asked to indicate which they preferred. This screen was self-paced. Each of the 42 affect-rich and the 42 affect-poor problems was presented twice, with each of the two options for each problem being presented once in the evaluation phase. The affect-rich and affect-poor problems were presented in alternating blocks of three problems from the same domain; within each domain, the problems were presented in randomized order. Participants entered responses via a button box, with the left button indicating that they chose the left option on the screen, and the right button, the right option. The duration of the fixation screens varied randomly between 2 and 9 seconds between the evaluation phase and the decision phase and between 3 and 11 seconds between the decision phase and the evaluation phase for the next trial.

The affective evaluation task was again completed outside the scanner; the order of presentation of the monetary amounts and the side effects was counterbalanced across participants. Overall, we employed a 2 × 2 design, with the domain of lottery problems (affect-poor vs. affect-rich) as a within-subjects factor and the ordering of affect-rich or affect-poor outcomes in the affective evaluation task (side effects vs. monetary losses) as a between-subjects factor.

### Computational Modeling

We used computational modeling to examine the cognitive mechanisms underlying participants’ decisions in the affect-rich versus affect-poor problems. Specifically, we modeled their choices using cumulative prospect theory (CPT) [14]. CPT assumes a transformation of objective outcomes into subjective values and of objective probabilities into decision weights. The degree of transformation in the *value function* and the *probability weighting function* is governed by free parameters, which are assumed to reflect sensitivity to outcome and probability information, respectively. CPT was fitted to each individual’s choices separately for affect-rich and affect-poor problems.

According to CPT, the subjective value of a negative outcome *x* (note that we only used negative outcomes) follows from the value function

$$v(x_{i})=-{\left(-x_{i}\right)}^{\alpha }.$$ (1)

The parameter α is constrained within the range [0,1], yielding a convex value function for losses (modeling diminishing sensitivity to outcomes). With outcomes 0 ≥ *x*_*1*_ ≥ … ≥ *x*_*k*_, and the corresponding probabilities *p*_1_ … *p*_k_, the weight π given to a negative outcome is the difference between the probability of receiving an outcome at least as bad as *x* and the probability of receiving an outcome worse than *x*:

$$\pi_{j}=w(p_{j}+\ldots p_{k})-w(p_{j+1}+\ldots p_{k}).$$ (2)

In Equation 2, *w*(*p*) is the probability weighting function, in which objective probabilities are transformed as follows:

$$w(p)=\frac{\delta p^{\gamma }}{\delta p^{\gamma }+{\left(1-p\right)}^{\gamma }}.$$ (3)

The parameter γ governs the sensitivity to differences in probabilities and is assumed to be in the range [0,1], with lower values yielding a more inverse S-shaped curvature of the function (indicating lower sensitivity). Note that, by way of the weighting function, CPT can thus represent perfect to strongly attenuated probability sensitivity. The parameter δ governs the elevation of the weighting function and can be interpreted as a measure of risk aversion, with higher values indicating more risk aversion (with δ > 0) [27, 28]. The overall valuation *V* of lottery *A* is determined as follows:

$$V(A)=\sum_{j=1}^{k}v(x_{j})\pi_{j}.$$ (4)

CPT predicts that the lottery with the more attractive *V* is more preferred.

To predict the probability *p*_*i*_(A, B) that lottery A is chosen over B in problem *i*, we used a choice rule that combines the softmax rule [29] and random guessing [24, 30]:

$$p(A,B)=(1-g)\frac{e^{V(A)}}{e^{V(A)}+e^{V(B)}}+\frac{g}{2},$$ (5)

where the parameter *g* represents the probability of guessing. Note that softmax is sometimes used with an additional parameter that governs how sensitively the predicted choice probability is to the difference in the subjective valuation of the lotteries [31]. We used the choice rule with only the guessing parameter, because the model fit (based on the Bayesian Information Criterion) [32] was better than that of a choice rule with both parameters or with only the choice sensitivity parameter.

To reflect the main assumptions of CPT, the parameter values were restricted as follows [31]: 0 < α ≤ 1; 0 < γ ≤ 1; 0 < δ ≤ 10; 0 < *g* ≤ 1. We fitted the model parameters to maximize the likelihood of the observed choices, using *G* ^*2*^ as index of fit (with smaller values indicating a better fit) [33]:

$$G^{2}=-2\sum_{i=1}^{N}\ln\left[f_{i}\left(y|\theta \right)\right],$$ (6)

where *N* is the total number of choices, and *f*(y|θ) is the probability with which the model with the set of parameter values θ predicts an individual’s choice *y*. That is, if lottery A was chosen, then *f*(y|θ) = *p*_*i*_(A,B) (with *p*_*i*_(A,B) defined as in Equation 5); if lottery B was chosen, then *f*(y|θ) = 1—*p*_*i*_(A,B). In the fitting procedure, we first implemented a grid search to identify the combinations of parameter values that minimized *G* ^*2*^. The 20 best-fitting value combinations were then used as starting points for subsequent optimization using the simplex method [34], as implemented in MATLAB.

### FMRI Data Acquisition

Functional MRI data were acquired on an Achieva 3 Tesla whole-body MR unit (Philips Medical Systems) using a standard echo planar imaging sequence with 39 axial slices of 3 mm (field of view 210 × 210 × 117 mm, 3 × 3 mm in-plane, repetition time 2 s, echo time 30 ms, flip angle 70°, SENSE factor 2). The field of view was rotated approx. E° relative to the AC-PC line to improve signal in the orbitofrontal cortex. Three runs of approx. 450 volumes were acquired (the exact number of volumes depended on the pace with which participants made their choices), as well as a high-resolution T1-weighted scan directly after the last fMRI sequence (repetition time 7.1 ms, echo time 3.2 ms, flip angle = 8°, field of view 256 × 256 mm, in-plane resolution 1 × 1 mm; slice thickness 1 mm [no gap], 160 axial slices).

### FMRI Data Analysis

#### Preprocessing

The fMRI analyses were performed using the FMRIB (Oxford Centre for Functional Magnetic Resonance Imaging of the Brain) Software Library (FSL) [35, 36]. Preprocessing of fMRI data involved slice-timing correction, motion correction (Gaussian kernel with full width at half maximum of 5 mm), temporal filtering (cutoff = 100 s), and denoising based on FSL melodic [37]. Denoising was performed as described in Tohka et al. [38]; we fitted decision thresholds for our study based on “true” signal and noise components manually identified for 20% (randomly chosen) of the runs. T1 images were skull-stripped using FSL’s Brain Extraction Tool (BET) [39].

#### Functional MRI analyses

FMRI analyses were first performed for individual runs (autocorrelation of residuals was reduced through pre-whitening). Statistical maps were first registered to each individual’s high resolution T1 image with FSL’s Boundary-Based registration (BBR) tool, and then to the Montreal Neurological Institute (MNI) standard space using linear and nonlinear transforms with FSL’s Linear Image Registration Tool (FLIRT) and Nonlinear Image Registration Tool (FNIRT) [40]. Statistical maps in standard space were averaged for each participant using a fixed effects analysis in FSL’s fMRI Expert Analysis Tool (FEAT). For one participant, the data of one run was missing; the average was therefore taken for the available two runs. The final group analysis was performed based on individuals’ averaged runs using FEAT’s flame 2 and automatic outlier detection [41].

FMRI data were analyzed using the general linear model (GLM) framework. Separate contrasts focused on either the evaluation or the decision phase. We nevertheless modeled the blood-oxygen-level dependent (BOLD) response for both phases in each analysis, because otherwise task-related activation in the phase that was not currently of interest would remain unmodeled and enter the error term. Each event was modeled by specifying its onset time, its duration (4 s for the evaluation phase and the response time for the decision phase), and a weight, which was 1 for intercept regressors and parametric for regressors modeling decision variables, such as loss magnitude or decision weights. In addition, a regressor for no-response trials and six motion regressors were included in the analysis. Finally, we included dummy regressors to capture variance due to sudden movements during scanning identified with the fsl_motion_outliers script. All reported results were tested for significance by first using a z-threshold of 2.3 (*p* = .005) and then a cluster-size threshold obtained using the AlphaSim tool from the AFNI (Analysis of Functional NeuroImaging) program [42]. The cluster-size thresholds were determined based on the volume of the entire brain. Exceptions were analyses regarding qualitative differences between brain BOLD responses, where the second test of contrasts within conjunctions used the conjunction volume to determine the cluster-size threshold. Further, small volume correction was used for the analysis of amygdala activity (the small volume was defined as voxels with a probability of at least 5% of being in the amygdala according to the Harvard–Oxford Probabilistic Atlas). The cluster-size thresholds are reported in the tables for the respective analyses.

### Comparison of affect-poor and affect-rich choices

In the first analyses, we tested for qualitative differences in brain activation between the affect-rich problems (choices between side effects) and the affect-poor problems (choices between monetary losses). The core design matrix implemented a factorial design, resulting in six regressors modeling the decision phase of each choice trial.

The six regressors in the decision phase were:

- intercept for overall activity during the decision between side effects (unmodulated);
- magnitude of the side effect of the chosen option (modulated);
- probability of the side effect of the chosen option (modulated);
- intercept for overall activity during the decision between monetary losses (unmodulated);
- magnitude of the monetary loss of the chosen option (modulated);
- probability of the monetary loss of the chosen option (modulated).

The modulated regressors were orthogonalized with respect to the respective unmodulated intercept regressors, but not with respect to other modulated regressors, as the latter is not recommended in the absence of strong theoretical reasons. All regressors were convolved with a double-gamma hemodynamic response function, and the first derivative of each convolved regressor was added to the model to account for variation in the peak time of the hemodynamic response function. Orthogonalization and convolution were conducted in the same way for all fMRI analyses. The same design was used to model the evaluation phase, except that here we used the magnitude and probability of the presented option.

#### Domain-general risk processing

To identify brain areas that are activated during risky choice in general, we conducted a conjunction analysis identifying regions that showed significant activation in both affect-rich and affect-poor choice during the decision phase.

#### Qualitative difference analysis

To investigate whether affect-poor and affect-rich choice recruit qualitatively different brain circuits, we tested for a region-by-condition interaction [43]. Again, we first identified regions that showed significant activation in both affect-rich and affect-poor choice, representing logical conjunction [44]; we then investigated whether there was a significant number of voxels within these regions that showed greater activation in affect-rich choice, whereas other voxels within these regions showed greater activation in affect-poor choice. As our focus was on the underlying choice mechanisms, we examined brain activation during the decision phase.

#### Neural correlates of differential probability processing

Previous research suggests that people are less sensitive to probability information in affect-rich than in affect-poor choice. To examine which regions are involved in the processing of probabilities, we tested in a final analysis which brain regions’ activity correlated with trial-by-trial decision weights estimated for each participant individually with CPT, separately for trials with affect-rich and affect-poor stimuli. The core design matrix implemented three regressors modeling the evaluation phase. Note that, in this analysis, the affect-rich and the affect-poor problems were modeled by the same regressors. The three regressors in the evaluation phase were:

- intercept for overall activity during presentation of the option (unmodulated);
- subjective value of the monetary loss/side effect of the presented option (modulated);
- decision weight for the monetary loss/side effect of the presented option (modulated).

The entries in the decision weight regressor were set to equal the decision weights for the respective outcome (see Equations 2 and 3), using the individually fitted CPT weighting function (with the respective parameters separately for the affect-rich and the affect-poor problems) and the probabilities of the respective choice option. The entries in the value regressor were set to the transformed magnitudes (according to CPT’s value function; Equation 1) using the individually fitted CPT parameters for affect-rich and affect-poor choices, respectively. In this analysis, we focused on brain activity during the evaluation phase. As only one lottery is presented in this phase, it is more straightforward to associate brain activation and option-specific information [26]. The same design was used to model the decision phase, except that here subjective values and decision weights of the chosen option—rather than the presented option—were used.

**Fig 2:**
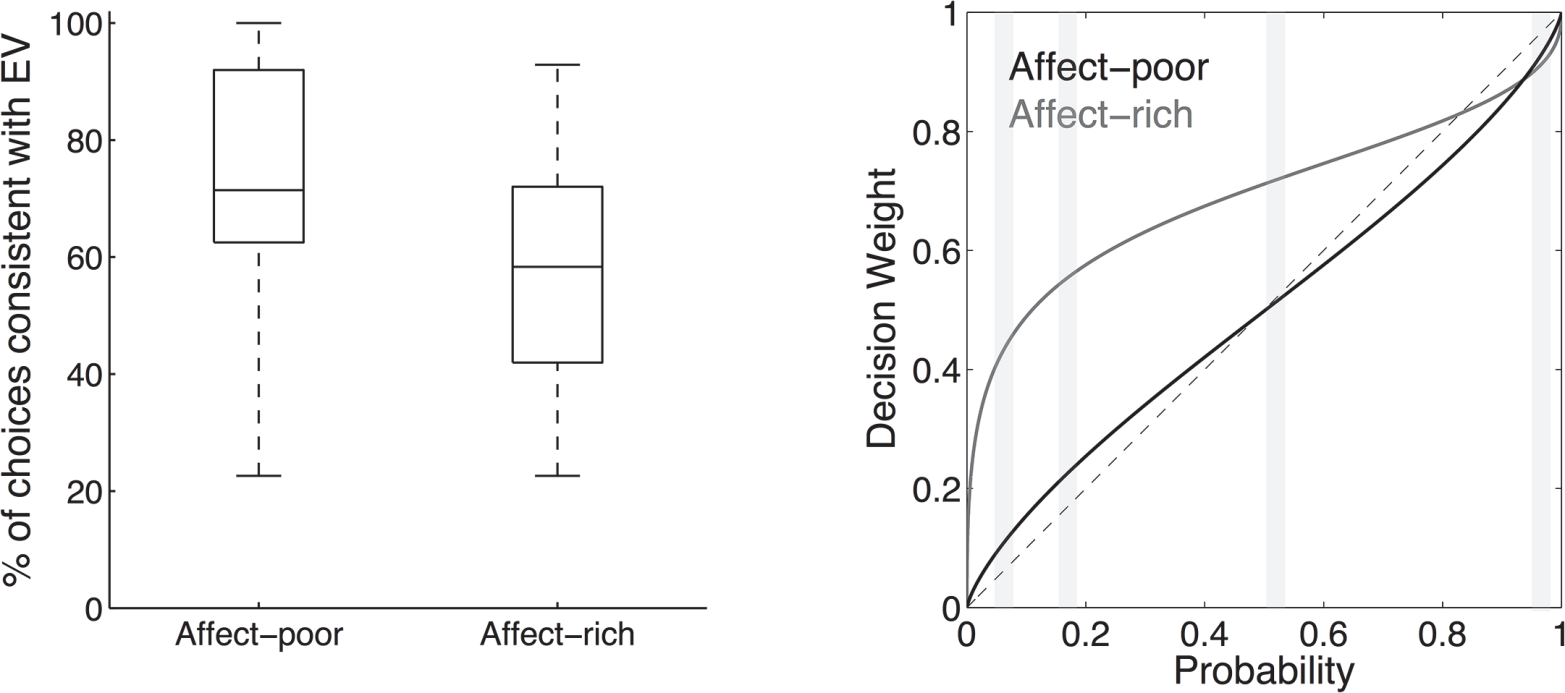
Expected Value Choices and Weighting Functions. (A) Median percentage of choices of the option with the higher expected value, separately for the affect-rich and the affect-poor problems. In each box, the central mark is the median, the edges of the box are the 25th and 75th percentiles, and the whiskers extend to the most extreme data points. (B) Cumulative prospect theory’s weighting function fitted to the choices in the affect-poor and affect-rich problems (here, using the average parameter values across all participants; Table 1). Probability levels used in the experiment are shaded. The dotted line indicates identity of probabilities and decision weights.

An alternative approach would be to model the decision weights with two separate regressors for the two domains, and then to contrast them against each other. However, we were mainly interested in identifying brain regions that showed little variation during affect-rich choice (as decision weights were rather similar across different probability levels; see Fig 2) and, at the same time, covaried with the more variable decision weights for affect-poor choice. We therefore chose a model that directly implements that assumption. Note that the observed effect cannot merely reflect greater activation in the affect-poor condition, as the average decision weight in this condition is lower than in the affect-rich condition (see Fig 2).

#### Posterior probabilities of terms given activation at a particular location

To establish the cognitive functions with which the identified regions are most strongly associated (based on previous studies), we conducted a formal reverse inference analysis, quantifying the association between brain activation and terms describing perceptual, emotional, cognitive, and motor functions. Our meta-analysis used the tools in the Neurosynth package, but extended the underlying list of terms and activation location databases (i.e., by adding terms from the Cognitive Atlas and including activation locations stored in the BrainMap database; S1 Appendix) [45–48]. The Neurosynth meta-analysis resulted in a list of terms (typically describing cognitive/psychological functions and processes) that have a high posterior probability given a contrast image and can be considered to provide an unbiased and data-driven picture of the cognitive processes associated with a contrast. Using the posterior probability to select terms ensures that only those terms are selected that are consistently associated with activation at a given location and, at the same time, that this location is rarely reported in articles in which the term is not mentioned. The analysis thus yields terms with high specificities.

## Results

### Behavioral Results

#### Affective evaluation

To assess whether experiencing a side effect was rated as evoking stronger negative affect than losing the equivalent monetary amount, we analyzed the ratings in the affective evaluation task using a 2 (domain: side effects vs. monetary losses) × 4 (outcomes) repeated-measures ANOVA (both factors were within-subjects). There was a significant main effect of domain, *F*(1, 22) = 7.01, *p* = .015, η^2^_p_ = .24, indicating that the side effects elicited more negative affect than did their monetary equivalents. This was the case for each of the four side effects. This finding supports our characterization of the medical side effects as affect-richer than the monetary losses.

#### Preference reversals

Were the differences in affect associated with different choices? Replicating previous studies [23, 24], our findings showed that, despite the monetary equivalence between affect-rich and affect-poor problems, people reversed their preferences between the corresponding problems in 46.07% (*SD* = 26.7) of cases, on average. An additional analysis using mixed-effects linear modeling (using the glmer function in the lme4 package in R, with “participants” and “problems” as random factors and “affect” as a fixed factor) [49] showed that participants selected the option with the higher expected value 2.05 times more often in the affect-poor than in the affect-rich choice, *b* = −0.72, *z* = −9.99, *p* < 0.001 (see also Fig 2A).

### Computational modeling

To examine the cognitive mechanisms underlying affect-rich and affect-poor choices, we modeled them using CPT. On average, CPT based on individually fitted parameters correctly described participants’ choices in 82.45% (*SD* = 12.39) of affect-rich choices and in 90.42% (*SD* = 6.74) of affect-poor choices. Fig 2B shows CPT’s probability weighting function based on the average best-fitting parameter value (Table 1). First, the probability weighting function was more strongly curved for affect-rich than for affect-poor choices, signaling lower sensitivity to probability information in the former than in the latter (as indicated by a lower value on the γ parameter of the weighting function). Furthermore, 82% (19 of 23) of participants showed lower probability sensitivity in affect-rich than in affect-poor choices. For 6 participants (26%), the γ parameter for affect-rich choice was very low (≤ 0.15), signaling almost complete insensitivity to probability information. Second, the probability weighting function was more elevated for affect-rich than for affect-poor choices, indicating higher risk aversion in the former.

**Table 1:** Average parameter estimates for cumulative prospect theory obtained in the affect-poor and affect-rich lottery problems and results of significance testing.

	Lottery Problems		Significance Test for Differences Between Affect-Rich and Affect-Poor Lottery Problems	
Parameters	Affect-Poor	Affect-Rich	*t*(22)	*p*	
γ	0.77 (0.24)	0.43 (0.33)	4.88	<. 001*	
δ	0.99 (1.63)	2.47 (3.10)	2.56	= .018**	
α	0.73 (0.21)	0.79 (0.26)	0.71	= .480	
*g*	0.07 (0.11)	0.12 (0.19)	1.15	= .261	
*G* ^*2*^	40.60 (23.86)	59.74 (32.81)	2.07	= .051	

*Note*. Standard deviations are in parentheses. γ and α model the sensitivity to probabilities and outcomes, respectively, with higher values indicating higher sensitivity; δ models the elevation, with higher values indicating higher risk aversion; *g* indicates the probability of random guessing. The *G* ^*2*^ expected under chance is 116.45.

* Significant tests after adopting a Bonferroni-Holm correction [50]. With *m* = 5 tests, the observed *p* values are first ordered in ascending order and are then tested with α_1_ = 0.05/*m*, α_2_ = 0.05/(*m*−1), …, α_*j*_ = 0.05/(*m*−(*j*−1)).

** One-tailed.

### FMRI Results

First, we identified basic decision making regions that showed significant activation in both affect-rich and affect-poor choices. Second, we investigated whether brain regions that are known to be involved in affective processing are differentially engaged during affect-rich versus affect-poor choices. Third, we tested for qualitative differences in task-dependent activation patterns in the affect-rich and affect-poor problems, which would suggest recruitment of qualitatively different decision mechanisms [43]. Fourth, as we did find task-dependent activation patterns, we examined which, if any, brain regions’ activity correlated with trial-by-trial decision weights (estimated for each participant individually with CPT, and separately for the affect-rich and affect-poor trials).

#### Domain-general risk processing

**Fig 3:**
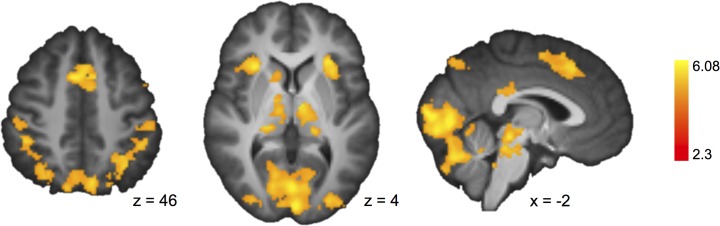
Brain Activation During Affect-Rich and Affect-Poor Choices. Among other regions, the occipital pole, thalamus, anterior insula and paracingulate gyrus showed greater activation in both decision making conditions than at baseline.

To investigate basic decision making regions, we identified brain areas that were activated during both affect-poor and affect-rich choice (Fig 3; Table 2). In addition to visual and association cortices, we found activation in regions typically recruited during risky choice, including the anterior insula, the thalamus, and the paracingulate gyrus [10].

**Table 2:** Regions activated during affect-rich and affect-poor choices. Clusters with k < 82 voxels are not shown.

Cluster	MNI Coordinates					k	Peak Significance	Anatomical Region
	X	Y		Z				
1	–2	–92	4		6617		6.75	Occipital pole, supracalcarine cortex, intracalcarine cortex
1	0	–76	0		–		6.62	Lingual gyrus, intracalcarine cortex
1	–40	–84	–10		–		6.4	Lateral occipital cortex, inferior division
2	24	–26	–4		1863		6.07	Right thalamus, right hippocampus
2	–8	–10	4		–		6.07	Left thalamus
2	0	–28	–12		–		6.02	Brain stem
3	42	–38	42		1169		5.84	Supramarginal gyrus, posterior division
3	–10	–70	44		–		5.83	Precuneous cortex
3	32	–66	44		–		5.64	Lateral occipital cortex, superior division
4	6	14	46		954		6.38	Paracingulate gyrus
4	–4	–2	56		–		5.35	Juxtapositional lobule cortex
4	6	28	40		–		5.18	Paracingulate gyrus
5	–36	–58	52		863		6.13	Superior parietal lobule, lateral occipital cortex, superior division, angular gyrus
5	–46	–40	44		–		5.74	Supramarginal gyrus, anterior division, supramarginal gyrus, posterior division, superior parietal lobule
5	–28	–50	42		–		5.38	Superior parietal lobule
6	30	22	4		442		6.21	Insular cortex
7	–42	8	36		330		6.42	Middle frontal gyrus
8	44	24	28		322		5.9	Middle frontal gyrus
8	48	38	22		–		4.95	Frontal pole, middle frontal gyrus
9	–28	22	0		294		6.17	Insular cortex
10	–46	30	20		207		5.87	Inferior frontal gyrus, pars triangularis, middle frontal gyrus
11	6	–38	26		202		5.36	Cingulate gyrus, posterior division
12	36	–66	–14		97		5.63	Occipital fusiform gyrus
13	–38	–8	64		87		5.6	Precentral gyrus

*Note*. Results are based on the Harvard–Oxford cortical and subcortical structural atlases.

### Differences in affective processing

**Fig 4:**
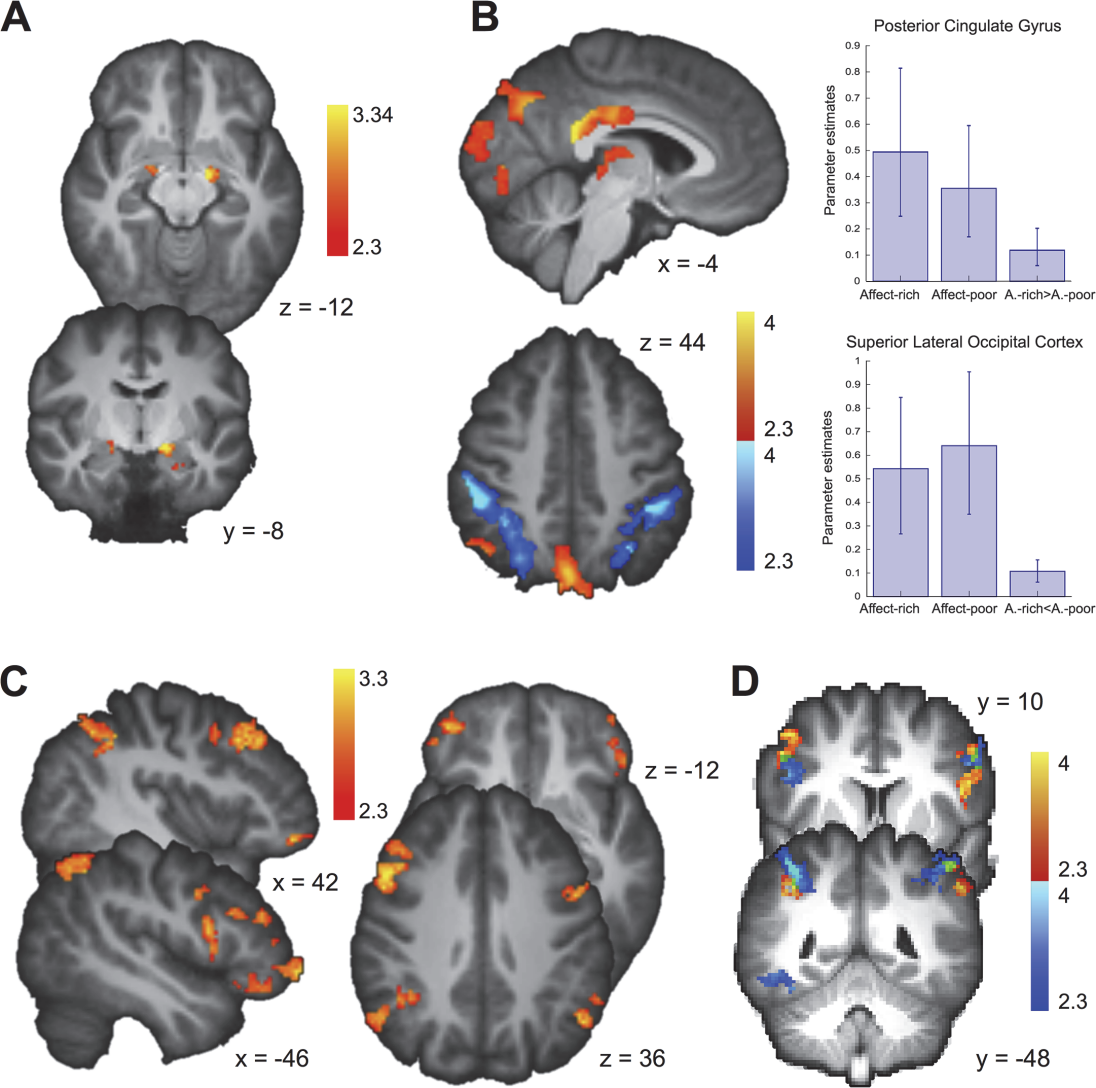
Differences in Brain Activation During Affect-Rich and Affect-Poor Choices. (A) Greater bilateral amygdala activation for affect-rich choices. (B) Region-by-condition interaction: Greater activation for affect-rich choices than for affect-poor choices in the posterior cingulate gyrus and the thalamus; greater bilateral activation for affect-poor choices than for affect-rich choices in the supramarginal gyrus and the superior lateral occipital cortex. Bar plots show percent signal change for contrasts versus baseline and for high-level contrasts, with error bars indicating 90% confidence intervals. (C) Among other regions, the supramarginal gyrus, the middle frontal gyrus, and the frontal pole were sensitive to the individually fitted decision weights. (D) Overlap (in green, displayed are the supramarginal gyrus and the middle frontal gyrus) of activation of affect-poor choice (displayed in B) and regions correlating with individually fitted decision weights (displayed in C).

**Fig 5:**
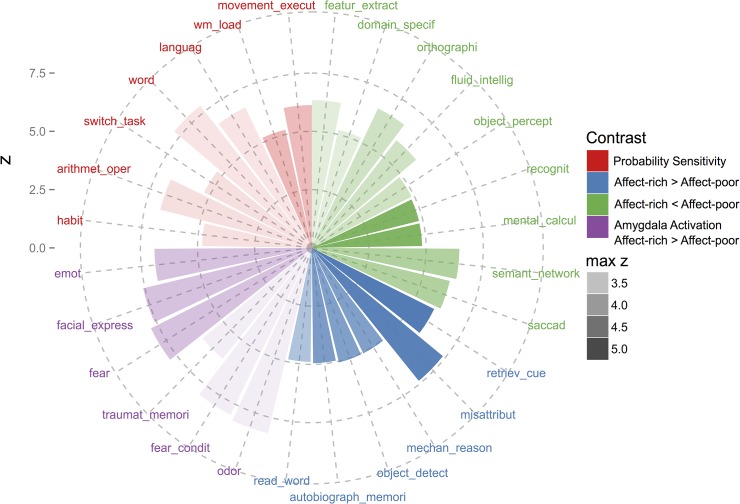
Polar Plot of Cognitive/Psychological Terms With a High Posterior Probability at Peak Locations of fMRI Analyses. To facilitate the interpretation of our results while respecting the limits to the validity of reverse inference based on fMRI data, we performed fMRI meta-analyses of 612 terms describing perceptual, emotional, cognitive, and motor functions based on peak locations reported in 7,500 neuroimaging articles. These meta-analyses resulted in posterior probability maps, where the value for each voxel is the probability that the process described by a term is recruited, given brain activation at this voxel (technically, it is the probability that an abstract or title of an article mentions the term, given activation). We used the posterior probability maps to find the terms with the highest posterior probability at peak locations from our fMRI analyses. The polar plot shows *z* values derived from Chi square tests of posterior probabilities. Different contrasts are represented by different colors, and the color transparency indicates the *z* value of the underlying fMRI contrast at peak location. The amygdala clusters with greater activation in the affect-rich domain (in purple) have high posterior probabilities for terms indicating emotional processes. Regions that are active in both domains, but to a greater extent during affect-rich choices (in blue), have higher posterior probabilities for introspective and memory processes. By comparison, regions more activated in affect-poor choices (in green) have a high posterior probability for mental calculations, but do not further cluster around a central topic. Finally, areas whose activity correlates with decision weights (in red) have high posterior probabilities for higher order cognitive functions, including arithmetic operations.

To the extent that (as hypothesized) choices concerning side effects imply greater emotional involvement than choices concerning monetary losses, stronger activation can be expected in brain areas involved in emotion processing. To test this hypothesis, we compared amygdala activation (during the decision phase) in the problems involving side effects relative to those involving monetary losses (overall activity during side effect > overall activity during monetary losses). We indeed found a stronger bilateral activation in the amygdala (Montreal Neurological Institute coordinates: X = −14, Y = −8, Z = −12; peak *z* = 3.71; 24, 6, −18; *z* = 3.14) during choice between affect-rich options than during choice between affect-poor options (Fig 4A; Table 3). To confirm that the activated regions were indeed good indicators of the processing of emotions, we conducted a formal reverse inference Neurosynth meta-analysis on the Neurosynth and BrainMap databases (for details, see Materials and Methods and S1 Appendix) [45, 46], quantifying the association between brain activation and terms describing perceptual, emotional, cognitive, and motor functions. More specifically, the Neurosynth meta-analysis yields a posterior probability that a previous study finding activation at a specific location also investigated a process or function described by the respective term. Following the approach implemented in the Neurosynth toolbox, we report *z* values from Chi-square tests performed on posterior probabilities (where higher posterior probabilities and higher term frequency lead to higher Chi-square and *z* values). Fig 5 shows that terms such as fear (*z* = 7.84), fear conditioning (*z* = 7.99), and emotion (*z* = 6.88) have a high posterior probability given the activation we observed in the amygdala.

**Table 3:** Greater bilateral amygdala activation for the affect-rich than for the affect-poor choices.

Cluster	MNI Coordinates			k	Peak Significance	Anatomical Region
	X	Y	Z			
1	–14	–8	–12	83	3.71	Left amygdala, left pallidum
1	–22	–6	–28	–	2.86	Parahippocampal gyrus, left anterior division hippocampus, left amygdala
2	24	6	–18	75	3.14	Frontal orbital cortex, right amygdala

Clusters with k < 23 voxels are not shown. *Note*. Results are based on the Harvard–Oxford cortical and subcortical structural atlases.

### Qualitative difference analysis

To examine choice-dependent brain activation (which would indicate qualitative differences in the cognitive processes underlying choices in the two kinds of tasks), we tested for a region-by-condition interaction in brain regions that were active during affect-poor and affect-rich choices, respectively (Fig 3) [43]. As our focus was on the underlying choice mechanisms, we examined brain activation during the decision phase. Fig 4B shows the identified region-by-condition interaction. Voxels in the posterior cingulate gyrus (MNI coordinates: X = −4, Y = −44, Z = 20; *z* = 3.14) and the thalamus (−4, −14, 8; *z* = 3.14) were active in both conditions, but more so in affect-rich than in affect-poor choice (Table 4). Voxels in the supramarginal gyrus (bilateral; 50, −36, 44; *z* = 4.84; −46, −40, 44; *z* = 4.75) and the superior lateral occipital cortex (bilateral; 30, −66, 30; *z* = 3.53; −30, −60, 44; *z* = 4.24), by contrast, were active in both conditions, but more so during affect-poor choice (Fig 4B; Table 5). The Neurosynth meta-analysis suggested that activations during affect-rich choice indicate recruitment of autobiographical memory (*z* = 4.95), whereas activations during affect-poor choice indicate recruitment of executive functions and calculative processing (*z* = 4.82; Fig 5).

**Table 4:** Regions with greater activation for the affect-rich than for the affect-poor choices.

Cluster	MNI Coordinates				k	Peak Significance	Anatomical Region
	X	Y	Z				
1	14	–94	–2	5688		5.14	Occipital pole
1	8	–68	32	–		4.69	Precuneous cortex, cuneal cortex
1	–14	–96	24	–		4.61	Occipital pole
2	–4	–44	20	725		5.24	Cingulate gyrus, posterior division
2	–2	–24	30	–		4.08	Cingulate gyrus, posterior division
2	6	–10	34	–		2.52	Cingulate gyrus, anterior division, cingulate gyrus, posterior division
3	22	60	2	206		3.76	Frontal pole
3	36	54	6	–		2.98	Frontal pole
4	46	–54	32	117		3.72	Angular gyrus
4	46	–64	50	–		2.88	Lateral occipital cortex, superior division
5	–2	–30	0	112		3.17	Brain stem, left thalamus
5	–4	–14	8	–		2.98	Left thalamus
6	–28	62	8	74		3.11	Frontal pole

Clusters with k < 65 voxels are not shown. *Note*. Results are based on the Harvard–Oxford cortical and subcortical structural atlases.

**Table 5:** Regions with greater activation for the affect-poor than for the affect-rich choices. Clusters with k < 65 voxels are not shown.

Cluster	MNI Coordinates					k	Peak Significance	Anatomical Region
	X	Y		Z				
1	50	–36	44		2164		4.84	Supramarginal gyrus, posterior division, supramarginal gyrus, anterior division
1	40	–48	52		–		4.83	Superior parietal lobule, angular gyrus
1	30	–66	30		–		3.53	Lateral occipital cortex, superior division
2	–46	–40	44		1577		4.75	Supramarginal gyrus, anterior division, supramarginal gyrus, posterior division, superior parietal lobule
2	–30	–60	44		–		4.24	Lateral occipital cortex, superior division, superior parietal lobule, angular gyrus
2	–28	–70	60		–		4.21	Lateral occipital cortex, superior division
3	44	–54	–10		361		5.01	Temporal occipital fusiform cortex, inferior temporal gyrus, temporo-occipital part,
3	36	–42	–22		–		3.04	Temporal occipital fusiform cortex, Temporal fusiform cortex, posterior division,
4	50	–74	–4		276		3.72	Lateral occipital cortex, inferior division
4	48	–80	10		–		2.95	Lateral occipital cortex, inferior division, lateral occipital cortex, superior division
4	32	–86	–2		–		2.65	Lateral occipital cortex, inferior division
5	–52	8	30		197		3.71	Precentral gyrus, inferior frontal gyrus, pars opercularis
6	48	4	20		191		4.45	Precentral gyrus, inferior frontal gyrus, pars opercularis
7	40	38	10		148		3.4	Frontal pole

*Note*. Results are based on the Harvard–Oxford cortical and subcortical structural atlases.

### Neural correlates of differential probability processing

According to the computational modeling analysis reported above, people are less sensitive to probability information in affect-rich than in affect-poor choice. Further, we found neuroimaging evidence for qualitative differences in task-related activations, with the regions activated in affect-poor choice being associated with more calculative processing. To examine whether these regions are indeed involved in probability processing, we implemented a model-based fMRI analysis. Specifically, we derived regressors for the fMRI analysis from the results of our computational modeling results (rather than using objective stimulus magnitudes) [51], and tested which brain regions’ activity correlated with trial-by-trial decision weights (which are derived from probabilities) estimated for each participant individually with CPT. In this analysis, we focused on brain activity during the evaluation phase, as it is more straightforward to associate brain activation with option-specific information when only one option is presented [26]. Brain activation in the supramarginal gyrus (bilateral; MNI coordinates: X = −58, Y = −44, Z = 42; *z* = 3.62; 38, −48, 40; *z* = 3.23), the middle frontal gyrus (bilateral; 50, 16, 36; *z* = 3.66; −42, 38, 20; *z* = 3.33), and the frontal pole (bilateral; 42, 48, −12; *z* = 3.23; −46, 56, −6; *z* = 3.23) correlated with the individual decision weights (Fig 4C; Table 6). A Neurosynth meta-analysis showed that activations correlating with the individual decision weights indicate recruitment of arithmetic operations (*z* = 6.83; Fig 5). These results suggest that the regions specifically recruited during affect-poor choice—but less so in affect-rich choice—indeed reflect processing of probability information (Fig 5).

**Table 6:** Regions that showed a positive correlation with the individual decision weight (derived from cumulative prospect theory) of the presented option.

Cluster	MNI Coordinates					k	Peak Significance	Anatomical Region
	X	Y		Z				
1	–58	–44	42		607		3.62	Supramarginal gyrus, posterior division, supramarginal gyrus, anterior division
1	–50	–58	38		–		3.45	Angular gyrus, lateral occipital cortex, superior division
1	–64	–30	44		–		2.9	Supramarginal gyrus, anterior division
2	50	16	36		594		3.66	Middle frontal gyrus
2	40	30	40		–		3.24	Middle frontal gyrus
2	50	6	52		–		2.8	Middle frontal gyrus, precentral gyrus
3	–42	38	20		518		3.33	Frontal pole, middle frontal gyrus
3	–42	8	32		–		3.2	Middle frontal gyrus, precentral gyrus, inferior frontal gyrus, pars opercularis
3	–52	14	4		–		3.14	Inferior frontal gyrus, pars opercularis
4	38	–48	40		346		3.23	Supramarginal gyrus, posterior division, superior parietal lobule, angular gyrus
4	44	–60	48		–		3.21	Lateral occipital cortex, superior division, angular gyrus
4	52	–56	30		–		3.1	Angular gyrus, lateral occipital cortex, superior division
5	–46	56	–6		189		3.23	Frontal pole
5	–50	30	–8		–		2.88	Frontal orbital cortex, inferior frontal gyrus, pars triangularis
5	–44	42	–16		–		2.85	Frontal pole
6	42	48	–12		104		3.23	Frontal pole
6	50	36	0		–		2.45	Frontal pole, inferior frontal gyrus, pars triangularis

Clusters with k < 82 voxels are not shown. *Note*. Results are based on the Harvard–Oxford cortical and subcortical structural atlases.

## Discussion

Numerous accounts of decision making under risk share the common notion that outcomes are weighted by their probability, with subsequent maximization of the (subjective) expected outcome. This study demonstrates that the notion of expectation maximization may not apply in an important domain of decision making under risk, namely in the context of prospects with emotionally laden outcomes. Taking advantage of a paradigm that allows us to compare between relatively affect-poor and affect-rich choices, we show that the two to some extent involve qualitatively different cognitive and brain mechanisms.

Specifically, we found systematically different preferences in affect-rich and affect-poor choice, with the option with the higher expected value being chosen considerably less often when outcomes were affect-rich than when they were affect-poor. Modeling individuals’ choices using CPT, we found affect-rich choice was best described by a substantially more strongly curved weighting function than affect-poor choice, signaling that the psychological impact of probability information is diminished in the context of emotionally laden outcomes. Put differently, probability information had a lesser impact on option evaluation in affect-rich choice. These results are in line with the findings of Pachur and colleagues [24], who showed that, in the context of affect-rich choice, participants seemed to avoid the option associated with the worse side effects, irrespective of their probabilities, and therefore often ended up choosing the option with the lower expected value.

Neuroimaging analyses further supported the hypothesis that choices between affect-rich options are based on qualitatively different cognitive processes than choices between affect-poor options; the two triggered qualitatively different brain circuits. Affect-rich problems engage more affective processing, as indicated by stronger activation in the amygdala. Furthermore, an examination of task-dependent brain activation revealed a region-by-condition interaction, which, according to Henson [43], is a stronger indication for the recruitment of separate brain mechanisms than is a double dissociation. To identify the psychological functions most likely recruited in the observed activation, we conducted a formal reverse inference meta-analysis on the Neurosynth and BrainMap databases, quantifying the association between brain activation and terms describing perceptual, emotional, cognitive, and motor functions. The results suggested that affect-poor choice is based on calculative processes, whereas affect-rich choice involves emotional processing and autobiographical memories.

Finally, using model-based neuroimaging analyses we examined whether the regions activated in affect-poor choice were in fact involved in the processing of probabilities. We found that brain activation in regions that were more active during affect-poor choice (e.g., the supramarginal gyrus and the middle frontal gyrus) correlated with individual trial-by-trial decision weights. These results suggest that the regions specifically recruited during affect-poor choice—but less so during affect-rich choice—indeed reflect processing of probability information.

In sum, our results imply that probabilities seem to impact decisions to a lesser degree in affect-rich than in affect-poor decision making. When a choice elicits strong emotions, decision makers seem to focus instead on the potential outcomes and the memories attached to them.

These findings have both theoretical and practical implications. On a theoretical level, models assuming expectation maximization (and implementing the weighting of some function of outcome by some function of probability) may fail to accurately predict people’s choices in the context of emotionally laden outcomes. Instead, alternative modeling frameworks (e.g., simplifying, lexicographic cognitive strategies) may be more appropriate [8, 52]. Our results thus also support objections to the dominant practice of investigating decision making under risk primarily in the context of monetary lotteries [53] and contribute to domain-specific theories of risky choice. These objections are not limited to behavioral research, but also pertain to neuroimaging research on risky choice. Inspection of recent review articles [54–57] and the 20 most cited neuroimaging articles on risky choice—identified on May 20^th^, 2014, on ISI Web of Science with the search string “(fmri or neuroimaging) and risk and (decision or choice)”—reveals that none of these articles considers whether or how the domain of the decision affects risk preferences. Instead, all of them use (different types of) lottery problems with monetary outcomes. Our finding of domain-specific decision mechanisms suggests that at least some insights into the brain mechanisms involved in decision making that have been obtained with relatively affect-poor monetary lottery problems do not easily generalize to the important class of choice domains that engage emotions, such as decisions about health [18, 23, 24] or, in some cases, even political decision making [19]. Our findings advance previous research demonstrating systematic preference reversals between affect-rich and affect-poor tasks [24, 17] by showing that different brain mechanisms are associated with affect-rich and affect-poor choice.

It is instructive to compare our results with a recent meta-analysis on neuroimaging experiments of risky choice. Using an activation likelihood estimation (ALE) meta-analysis, Mohr et al. [10] found higher involvement of the right anterior insula, dorsomedial prefrontal cortex, dorsolateral prefrontal cortex, and parietal cortex specifically during actual choice relative to only the anticipation of risky outcomes. To the extent that greater activation of these regions reflects processing of probabilities and outcomes during choices between monetary lotteries (the task used in nearly all studies in the meta-analysis), one might expect them to show greater activation during affect-poor than affect-rich choice in the present experiment. In fact, we found most of these regions to be involved in both (Fig 3; Table 2), but that they were not differently activated for each kind of choice. One potential reason is that while the research summarized by Mohr et al. used lottery problems with only positive or mixed outcomes, our lottery problems involved only losses. It is unclear to date whether negative and positive risky outcomes are processed in the same regions. An alternative possibility is that the regions reported by Mohr et al.—though consistently activated during risky choice—are not specific to risky choice. This interpretation is supported by our finding that none of the regions reported in Mohr et al.’s analysis were identified in a Neurosynth analysis as having a high posterior probability (which would indicate processing specific to risky choice). More generally, comparison of our results with summaries of previous neuroimaging studies on risky choice highlights that past research focused predominantly on monetary lotteries and thus on only a narrow sample of decision making tasks involving risk. Moreover, the challenge remains to identify regions that are specifically involved in risk processing, as opposed to regions that are generally involved in decision making.

Although Neurosynth analyses provide unbiased reverse-inference results, it should be noted that the strength of the conclusions drawn from a Neurosynth analysis is limited by the quality of the underlying data. In particular, Neurosynth analyses cover a good part but not all of the neuroimaging literature, are based on peak coordinates and not complete activation images, and do not include information about specific contrasts. Despite these limitations, Neurosynth results for broad cognitive and motivational categories have been found to be consistent with results from other meta-analyses based on more detailed information about experiments and contrasts (e.g., the BrainMap database) [46, 47]. Hence, the reverse inference results reported here can be understood as a first exploration of the differences in cognitive processes that are driven by different brain activation patterns.

As mentioned above, the study of the role of emotions in decision making has a long history in behavioral and neuroscientific inquiries into risky choice, as evidenced by theories of anticipated regret [58–61] and the “risk as feelings” hypothesis [15, 54]. In our view, this approach is orthogonal to our treatment of the role of affect for decision making. Most of these theories were developed as alternatives to traditional accounts of decision making under risk and are thought to describe risky choice independent of decision domain. Our results complement these theoretical approaches by underlining the importance of emotions in decision making: domains that are more likely to engage emotions may trigger fundamentally different brain mechanisms than domains that are devoid of or less rich in emotions.

Further, to the extent that people show strongly attenuated sensitivity to probability information (or even neglect it altogether) in decisions with affect-rich outcomes, different decision aids may be required to help them make good choices. For instance, professionals who communicate risks, such as doctors or policy makers, may need to pay special attention to refocusing people’s attention on the probabilities of (health) risks by illustrating those risks visually [62, 63].

## Conclusion

We compared choice in relatively affect-poor monetary lottery problems with choice in problems that engage more affective processing. Computational modeling of the behavioral data and model-based neuroimaging analyses produced converging evidence that affect-rich and affect-poor risky choice to some extent recruit qualitatively different decision mechanisms, on both the cognitive and the neurobiological level. Whereas affect-poor choice was sensitive to probability and recruited brain regions indicative of cognitive and number processing, affect-rich choice was relatively insensitive to probability and recruited brain regions involved in processing emotions and autobiographical memories.

## Supporting Information

S1 Table Lottery Problems. (PDF)

S1 Appendix Posterior Probabilities of Terms Given Activation at a Particular Location. (PDF)
