# Supplementary material for: The Neural Basis of Risky Choice with Affective Outcomes
Source: PLoS One. 2015 Apr 1;10(4):e0122475. doi: 10.1371/journal.pone.0122475 (PMC4382171; doi:10.1371/journal.pone.0122475)
Supplement: S1 Table — (PDF) [file pone.0122475.s001.pdf]

Table S1.
Lottery problems in the affect-rich task and each side effect's median monetary evaluation (obtained in a pilot study in Switzerland, therefore indicating monetary evaluations in Swiss Francs), on the basis of which the problems were constructed. For the problems in the affect-rich task, the side effects were inserted according to each participant's individual rank order. For the problems in the affect-poor task, they were replaced with each participant's individual monetary evaluation of the respective side effect. All 42 lottery problems were presented twice.

Lottery A			Lottery B		
Probability	Side Effect (Rank)	*Mdn* (From Pilot Study)	Probability	Side Effect (Rank)	*Mdn* (From Pilot Study)
0.95	3	18	0.53	2	30
0.18	3	18	0.08	1	50
0.05	1	50	0.18	2	30
0.05	1	50	0.18	3	18
0.50	1	50	0.95	2	30
0.53	3	18	0.18	2	30
0.95	4	10	0.18	2	30
0.98	2	30	0.50	1	50
0.18	3	18	0.08	2	30
0.18	1	50	0.98	4	10
0.08	1	50	0.5	4	10
0.15	3	18	0.53	4	10
0.95	4	10	0.18	2	30
0.15	2	30	0.98	4	10
0.05	3	18	0.15	4	10
0.95	4	10	0.08	1	50
0.08	1	50	0.18	2	30
0.18	4	10	0.05	2	30
0.98	3	18	0.50	1	50
0.95	3	18	0.15	1	50
0.53	4	10	0.08	2	30
0.98	4	10	0.08	1	50
0.53	4	10	0.05	1	50
0.95	3	18	0.50	2	30
0.18	4	10	0.05	1	50
0.08	2	30	0.50	4	10
0.15	2	30	0.53	4	10
0.15	1	50	0.50	3	18
0.50	2	30	0.15	1	50
0.95	4	10	0.15	1	50
0.05	2	30	0.15	3	18
0.18	4	10	0.05	3	18
0.98	3	18	0.53	2	30
0.08	3	18	0.15	4	10
0.08	1	50	0.5	3	18
0.98	2	30	0.53	1	50
0.95	4	10	0.50	3	18
0.95	4	10	0.08	1	50
0.95	3	18	0.18	1	50
0.08	2	30	0.15	3	18
0.53	3	18	0.98	4	10
0.50	2	30	0.98	3	18

Note. Side effect (Rank) refers to each participant's individual rank order, with 1 indicating the side effect that was ranked as most unpleasant and 4 the effect ranked as least unpleasant. Side effects used were fatigue, insomnia, depression, and memory problems. As participants differed in terms of their monetary evaluations of the side effects, the monetary losses in the affect-poor problems varied between participants.
