# Supplementary material for: The Neural Basis of Risky Choice with Affective Outcomes
Source: PLoS One. 2015 Apr 1;10(4):e0122475. doi: 10.1371/journal.pone.0122475 (PMC4382171; doi:10.1371/journal.pone.0122475)
Supplement: S1 Appendix — (PDF) [file pone.0122475.s002.pdf]

Posterior Probabilities of Terms Given Activation at a Particular Location

To establish the cognitive functions in which the identified regions are most involved, we conducted a formal reverse inference analysis, quantifying the association between brain activation and terms describing perceptual, emotional, cognitive, and motor functions. Terms were single- or two-word combinations that authors used in their articles, and can thus be assumed to describe the function investigated. Our meta-analysis used the tools in the NeuroSynth package, but extended the underlying list of terms and activation location databases. We extended the list of terms because (a) the original NeuroSynth list contains only single-word terms, whereas two-word terms are often more informative; (b) the NeuroSynth database treats different forms of the same word (e.g., plural and singular, past and present forms) as different terms, whereas we used word-stems to avoid this; and (c) the NeuroSynth word list is sourced from word frequencies in articles without systematic consideration of the accumulated knowledge about types of (cognitive) functions, whereas we extended this body of knowledge by adding terms from the Cognitive Atlas [43].

To better reflect the current literature, we expanded the dataset used for our meta-analysis by including activation locations stored in the BrainMap database [44]. Because articles are manually entered in this database, it contains more specific data (i.e., clear descriptions of contrasts associated with locations) than the NeuroSynth database. On the other hand, it contains data from fewer articles (2,390 in BrainMap vs. 5,900 in NeuroSynth) and allows meta-analyses only for relative broad areas of functioning. Combining the locations from the BrainMap and NeuroSynth databases resulted in a new location database with locations from 7,500 unique articles (i.e., an increase of about 25% relative to the original NeuroSynth database).

Expanding the Neurosynth location database necessitated extraction of terms mentioned in all papers in a consistent manner. Two general approaches can be used to distinguish relevant terms (i.e., those describing the topic of an article) from irrelevant ones.

First, one can check the frequency of every word used in an article and define relevant words as those exceeding a threshold (NeuroSynth uses 0.1%). Second, one can assume that all words in title, abstract, and keywords describe the topic of an article, so that the occurrence of a term in these fields indicates that the paper indeed investigated the function described by that term. As we see no strong arguments to prefer either method, and because the second method is faster to implement (i.e., in most cases, it requires only access to Pubmed, whereas the first requires full text access to all articles), we used the second method.

To calculate posterior probabilities of terms given the observed activations as described by Yarkoni and colleagues [46] we conducted the following steps:

1) As described above, we generated a list of terms describing cognitive and affective processes by (a) starting with the terms used in the NeuroSynth database (https://github.com/neurosynth/neurosynth-data/blob/master/features.txt), (b) adding one- and two-word terms found in the Cognitive Atlas (http://www.cognitiveatlas.org/concepts/a), and (c) stemming all words in the term list and removing stop words (e.g., "of," "by," "end") with the natural language toolkit (http://nltk.org).

2) We compiled a database with activation locations indexed by Pubmed IDs for all articles in the NeuroSynth (https://github.com/neurosynth/neurosynth-data/blob/master/features.txt, retrieved November 15th 2013) and in the BrainMap databases (http://www.brainmap.org, retrieved with "Sleuth" on November 15th 2013). This new, combined database comprises activation locations for 7,500 articles.

3) We compiled a new feature database by retrieving title, abstract, and keywords for each article, concatenating these strings, stemming and removing stop words, and testing for occurrence of terms from our term list in the concatenated string (i.e., at least one occurrence in either the title, abstract, or keywords of an article). All terms that occurred in fewer than 15 articles as well as the redundant or overly general terms "face*", "house", "picture", "actor" were removed from the resulting database.

4) We used the NeuroSynth toolbox (https://github.com/neurosynth/) to calculate the posterior probability of a term given activation at a location.

The posterior probability is defined as

$$p(Term|Actv.) = \frac{p(Actv.|Term) * p(Term)}{p(Actv.|Term) * p(Term) + p(Actv.|notTerm) * (1 - p(Term))} \qquad \text{(S1)}$$

Using the posterior probability to select terms ensures that only those terms are selected that are consistently associated with activation at a given location and that at the same time this location is rarely activated in articles not mentioning the term. Put differently, focusing on high posterior probabilities focuses the interpretation of activation on terms with high specificities.

Because cognitive processes are often implemented in a distributed manner and multiple processes can influence decision making in our task, we identified multiple peak locations for each contrast. Peak locations and associated terms were identified as follows:

a) Within each cluster, we identified local maxima using FSL's cluster command (min distance between local maxima: 3cm).

b) For each location (local maximum in a cluster), we created a region of interest (ROI) as a sphere with 5 mm radius around the location and calculated the average posterior z-value for each term for the ROI. Specifically, posterior z-values were calculated within the NeuroSynth meta-analysis from chi-square statistics on posterior probabilities, such that the number of articles mentioning a term as well as the posterior probability influences the z statistic. Average posterior z-values for a term and ROI were calculated as weighted means of posterior z-values, using the z-statistic of our underlying fMRI contrast (normalized so that they summed to 1) as weights.

c) To extract the most relevant cognitive terms, we calculated an "evidence score" by multiplying the peak z-value from our fMRI contrast in each ROI with the average z-value for the posterior probability of each term in same ROI. We then extracted for each contrast the

eight terms with the highest evidence score. When contrasts had only a single cluster, we extracted for each cluster the three terms with the highest evidence score.

d) If a term was associated with multiple peak locations within a contrast, only the highest z-value for this term was extracted.

This procedure resulted in a list of terms that have a high posterior probability given the contrast image and can be considered to provide an unbiased and data-driven picture of the cognitive processes associated with a contrast.
